# Nano and micro architectures for self-propelled motors

**DOI:** 10.1088/1468-6996/16/1/014802

**Published:** 2015-01-28

**Authors:** Jemish Parmar, Xing Ma, Jaideep Katuri, Juliane Simmchen, Morgan M Stanton, Carolina Trichet-Paredes, Lluís Soler, Samuel Sanchez

**Affiliations:** Max Planck Institute for Intelligent Systems, Stuttgart, Germany, Heisenbergstr. 3, 70569 Stuttgart, Germany

**Keywords:** nanomotors, self-propellers, microfabrication, nanomachines, 3D printing

## Abstract

Self-propelled micromotors are emerging as important tools that help us understand the fundamentals of motion at the microscale and the nanoscale. Development of the motors for various biomedical and environmental applications is being pursued. Multiple fabrication methods can be used to construct the geometries of different sizes of motors. Here, we present an overview of appropriate methods of fabrication according to both size and shape requirements and the concept of guiding the catalytic motors within the confines of wall. Micromotors have also been incorporated with biological systems for a new type of fabrication method for bioinspired hybrid motors using three-dimensional (3D) printing technology. The 3D printed hybrid and bioinspired motors can be propelled by using ultrasound or live cells, offering a more biocompatible approach when compared to traditional catalytic motors.

## 1. Introduction

Self-propelled motors are still a very new topic of research, first demonstrated nearly a decade ago [1, 2]. Micromotors and nanomotors have piqued the interest of the scientific community in recent years, and various applications have been demonstrated so far [3]. Many biomedical applications of catalytic motors, such as biosensing [4], drug delivery [5], and nano- and micropumps [6] require a size range in the nanometer scale; other applications, such as cell transport [7, 8], cargo transport [9], and the drilling of biomaterials [10] require sizes in the micrometer range. In addition, micromotors have been demonstrated for use in both environment remediation [11] and motor-assisted lithography [12]. Hybrid microbiorobots are fabricated in the micrometer-to-millimeter scale, depending on the size of the mechanical force generating the biological material, such as a bacterial cell or a mammalian cell. Millimeter-scale catalytic motors [13] and centimeter-scale catalytic motors have been demonstrated previously [14]. Different sizes and shapes are important, both for use in different applications and also to understand the fundamental science related to motion at the nanoscale and microscale.

In this work, we present various types of architectures to fabricate different sizes and shapes of catalytic micro- and nanomotors that we are currently working on in our group. Detailed mechanisms of the motion of micro- and nanomotors are out of the scope of this article, and they have already been reported elsewhere [15–19]. We aim to provide an overview of the different fabrication strategies to help select the proper microfabrication and nanofabrication techniques, depending on the available facilities and the shape and size requirements of the motors.

## 2. Fabrication of motors

### 2.1. Micromotors from spherical architecture

In 2005, Golestanian *et al* proposed the first model for the reaction-driven propulsion of a micro-object through a diffusiophoretic mechanism [20], which was later proven experimentally for artificial catalytic Janus particles [21]. Since then, spherical Janus particles have played an important role in the field of micro/nanomotors. A variety of methods can be used to fabricate Janus particles with different materials, such as polystyrene [21–23], silica [24, 25], and catalytic or reactive metals [26–29]. Geometrically asymmetric Janus micromotors, such as ‘coconut’ micromotors and nanoshell micromotors, have been reported to achieve self-propulsion [30, 31]. At present, considerable research about spherical Janus motors has focused on the microsize range, as real ballistic motion can only be seen above 800 nm; below that value, the persistence length is too low to get any significant directional motion. There are limited reports [5, 32] about spherical Janus motors within the nanoscale where strong Brownian motion and fluid viscosity dramatically affects the motion of the motors. However, taking into account the significant role of nanosized materials in fundamental science research and practical applications, it is important to research spherical Janus nanomotors. The fundamental mechanism behind the motion of the Janus particles is still controversial. In general, a chemical reaction at only one side of the Janus particles produces a driving force for the motion of these motors. Janus motors driven by bubble propulsion [26–29, 33–35] are understood relatively clearly. However, without obvious bubble generation, different propulsion mechanisms have been proposed, including electrolyte/ionic self-diffusiophoresis [21, 36, 37] and self-electrophoresis [23, 32]. One of the advantages of using spherical microparticles is that they are self-propelled objects with inherent simplicity in their geometry, which facilitates the coordination between theory and experiment. It was shown that size influences motor speed [22] and geometry and motor design can influence their trajectories. Gibbs and Zhao presented the deposition of a TiO_2_ arm on self-propelled Janus particles, conferring additional torque to the motor and resulting in different motion types depending on the position of the catalyst [25]. We are interested in the fabrication of various sizes of micro- and nanospherical motors to address fundamental aspects of motion, and also to develop different applications.

#### 2.1.1. Nano- to microscale spherical motor fabrication method

We fabricated spherical Janus motors, based on solid silica (SiO_2_), with controllable sizes ranging from 125 nm to 650 nm. First, the solid silica spheres were prepared by a modified Stöber [38] method. Particle size was controlled by the concentration of ammonia used in the reaction. Typically, particle size increases with an increase in ammonia concentration.

To make Janus motors, first a monolayer of the solid silica spheres was obtained by drop-casting. The silica spheres were suspended in ethanol with a known concentration and then dropped onto clean, hydrophilic glass slides that were pretreated with oxygen plasma. Monolayers of commercial microscale silica and polystyrene spheres were obtained by drop-casting; alternatively, a more closely packed monolayer can be produced by using the Langmuir–Blodgett technique. Briefly, functionalized silica particles accumulated in the water at the interface of a binary system of a chloroform:ethanol (80:20) mixture. After solvent evaporation, the monolayer was located on the air-water interface, and was transferred onto a silicon wafer. In the following step, the prepared monolayer was placed in a homemade e-beam setup to deposit the catalytic layer of Pt under vacuum conditions. A few nm of titanium can be deposited to increase the stickiness of the particle surface before the deposition of a platinum layer for the microscale spheres.

**Figure 1.**
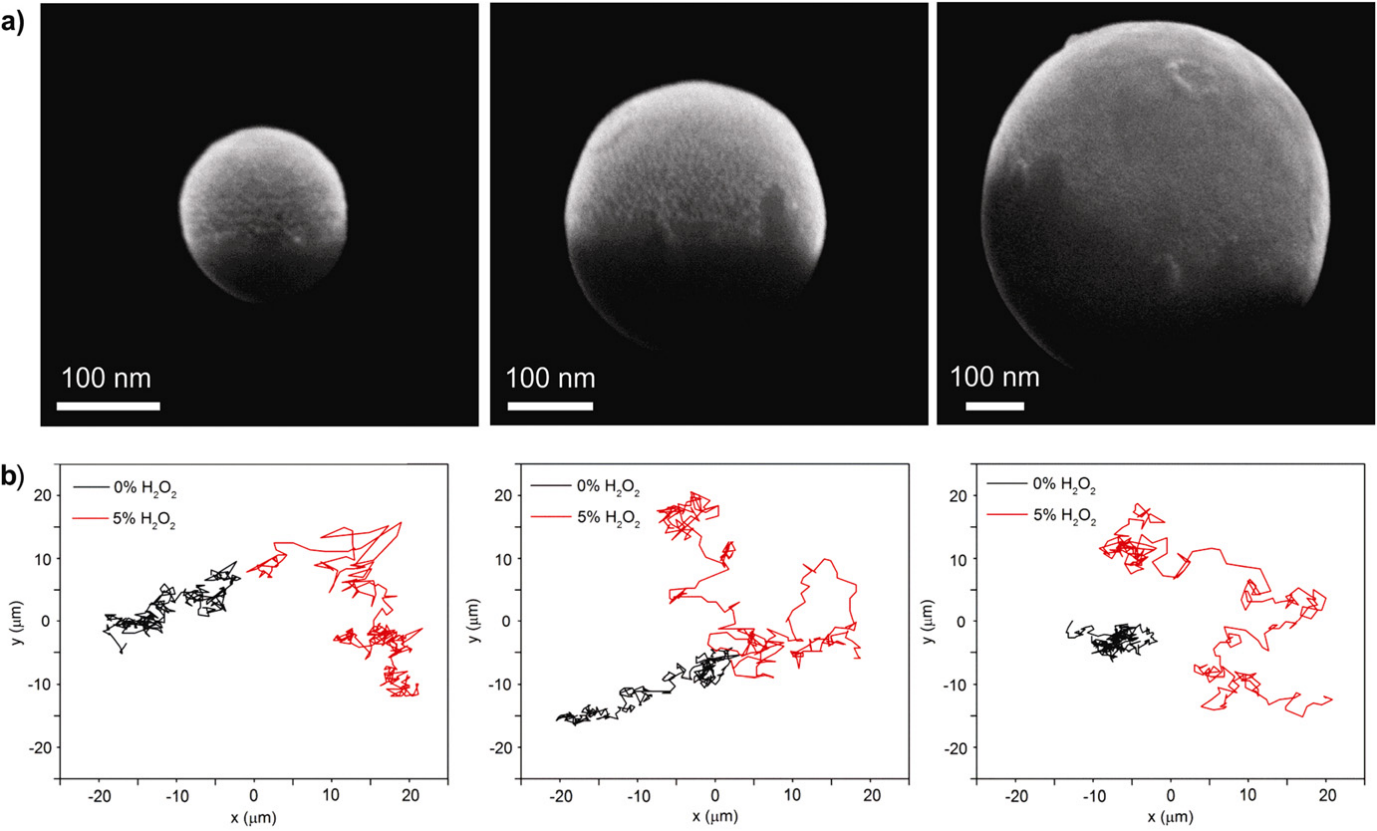
Fabrication and motion of Janus SiO_2_ nanospheres. (a) Scanning electron microscope images of a Janus SiO_2_ nanosphere with Pt (3 nm), and (b) their trajectory tracking, both with and without H_2_O_2_ fuel. Left to right: Janus SiO_2_ nanospheres of 125 nm, 330 nm, and 650 nm.

The obtained Janus spheres are collected by sonication and are then suspended in distilled water. The scanning electron microscopy (SEM) images of figure 1(a) show that three different sizes of spherical Janus particles half-coated with Pt were successfully obtained. The motion of these Janus nanoparticles was observed by optical microscopy, and their trajectories were tracked by ImageJ software (figure 1(b)). With a decrease in size, the influence of Brownian force becomes stronger, as indicated by the black trajectories without any presence of H_2_O_2_. However, with the addition of H_2_O_2_ fuel, the random motion of the Janus nanoparticles covers a broader range, as indicated by the red trajectories, suggesting self-propulsion of these Janus nanomotors, driven by catalytic reaction. According to previous research on microsized spherical Janus motors, such active motion is highly possible due to the self-diffusiophoresis mechanism that results from the solute gradient by the Pt-triggered decomposition of H_2_O_2_ on only one side of the Janus particles. Further research will focus on how to realize effective guidance of the self-propelling Janus nanomotors, allowing them to achieve directional motion in a controlled manner.

#### 2.1.2. Micromotor guidance by wall

Many approaches have been used to guide motors, including magnetic fields [19, 28, 39–44], gravitaxis [45], and chemotaxis [41, 46, 47]. Chemotaxis toward molecules other than fuel has rarely been observed, and pH-taxis has only been proven by Dey *et al* [48]. Our group wants to follow a biologically inspired approach: motor proteins such as myosin, kinesin, and dynein are small biomolecules responsible for most inner cellular transport. Generally objects smaller than 800 nm are considered unable to undergo directional motion because the influence of rotational diffusion (*kt*) affects their path. Nature overcomes this problem by using a microtubule track, along which motor proteins can ‘walk’.

**Figure 2.**
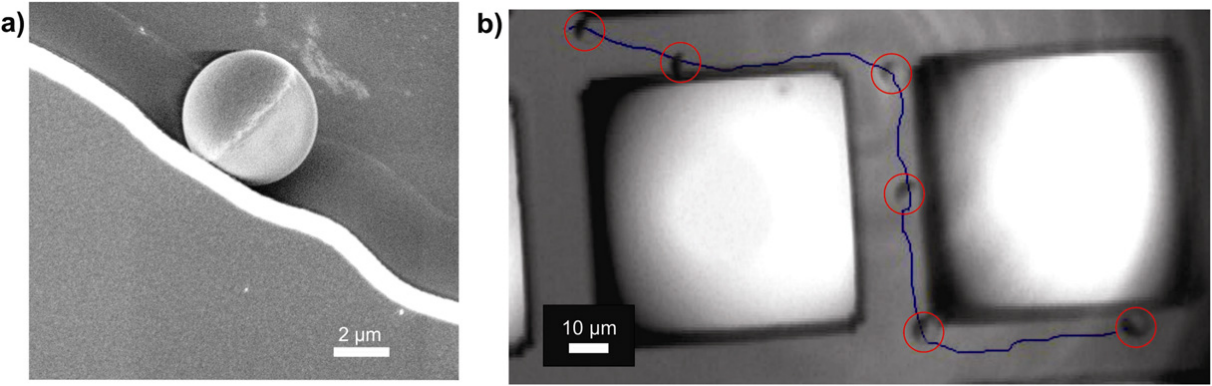
Wall guiding of Janus micromotors. (a) SEM image of Pt-capped Janus particle (5 *μ*m diameter) close to the 1 *μ*m-high glass wall, and (b) the trajectory, along with the wall they are following.

To achieve force-free particle guidance, we adopted the wall-guiding strategy and designed the walls to guide the particle’s motion. An obvious approach is the use of high walls that limit the fluid flow, and therefore restrict the accessible area for the micromotors, as shown by Baraban *et al* for particles and tubes [47]; the same group achieved active micromotor trapping in microfluidic chips [49]. We observed that particles feel walls and steps, and preferentially move along the walls until they encounter an obstacle or another stimulus. To design these walls, we considered the existence of an attraction potential between the particle and the wall, so that the particle cannot escape due to rotational diffusion. Figure 2 shows an example of Pt-capped Janus particles (5 *μ*m in diameter) that approach a 1 *μ*m-high glass wall and follow it. In some of the experiments, the particle even overcame a gap between two walls without significant deviation. An SEM image gives a more detailed view of the particle close to the wall architecture. Further work on this topic is underway in our laboratory.

### 2.2. Micromotors from tubular architecture

In 2011, Gao *et al* proposed an electrochemical method for the fabrication of catalytic microjets [50]. This method had a number of advantages over the conventional roll-up technology for microjets, which was developed by Mei *et al* in 2008 [51]. Electrodeposition only requires a potentiostat and a porous membrane, eliminating the need for a clean room and an electron beam evaporator, thus substantially reducing the cost of production. This method also does not have the size limitation that roll-up microjets have. So far, the smallest reported tubes produced by roll-up technology are at least 25 *μ*m in length. In contrast, the electrodeposited tubes can be fabricated in sizes as small as 2–5 *μ*m. As an advantage over electrodeposited micromotors, roll-up technology enables us to fabricate micromotors comprised of different materials that can be evaporated on a sacrificial layer. Micromotors a few hundred microns in length that are made of nonconductive metal oxides or polymers can be fabricated with this technology, which is generally not possible with the electrochemical method [52, 53].

**Figure 3.**
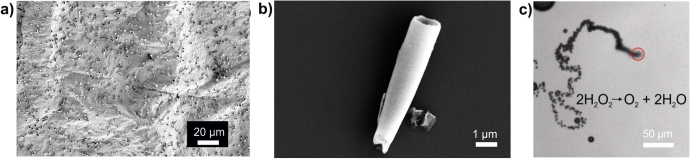
Fabrication and self-propulsion of electrochemical microjets. (a) SEM image of PEDOT-Pt tubes grown on gold film (the polycarbonate membrane is dissolved). (b) SEM image of a single tube. (c) PEDOT-Pt tubes in H_2_O_2_ that are propelled due to continuous bubble release. The trail of bubbles is clearly visible. Red circles indicate the tubes.

#### 2.2.1. Electrochemically fabricated tubular micromotors

The electrodepositing procedure detailed in [50] involves the use of a polycarbonate membrane with 2 *μ*m-diameter pores to guide the growth of the tubes. This membrane is coated on one side with 75 nm of gold, which acts as a working electrode. An Ag/AgCl electrode is used as a reference, and a platinum wire serves as the counterelectrode. First, a polymer layer is deposited from a solution containing 15 mM EDOT, 7.5 mM KNO_3,_ and 100 mM sodium dodecyl sulfate. Poly(3,4-ethylenedioxythiophene) (PEDOT), which is a conducting polymer, serves as the outer layer of these microtubes, and subsequent layers of metals are deposited on the PEDOT layer. Owing to the solvophobic and electrostatic effects [54], the PEDOT preferentially grows along the surface of the membrane and not as a rod in the pores. To make these tubes catalytically active, a layer of platinum is deposited on the PEDOT from a platinum-plating solution. After this, the gold layer is scratched off by hand polishing against alumina slurry, and the membrane is dissolved in a methylene chloride solution (figure 3(a)). The suspended PEDOT/Pt tubes are subsequently washed in ethanol and collected and stored in water. Figure 3(b) shows a single PEDOT/Pt microtube.

The tubes fabricated by this method exhibit a relative speed (body length per second) much larger than that observed in roll-up tubes for the same concentration of H_2_O_2_. Figure 3(c) shows a PEDOT/Pt micromotor self-propelling in the H_2_O_2_ fuel. Gao *et al* reported speeds up to 2400 *μ*m s^−1^ in 5% H_2_O_2_ for tubes produced by this method [55]. The mechanism of motion in these tubes remains the same as that of the larger roll-up tubes: bubble propulsion. Another advantage of these tubes is that they remain active over longer periods of time. After one hour, most of these tubes were found to be active, albeit exhibiting lower propulsion speeds. The mechanism of the microjet was reported elsewhere [18, 19, 56].

Additional directional control of these tubes can be achieved by adding an intermediate nickel layer between the polymer and the Pt. Because of the conical shape of the pores in the polycarbonate membrane, these tubes have one end slightly larger than the other. This allows for the preferential selection of the opening for bubble release. In the roll-up process, we rely on chance for one end to be slightly larger than the other to achieve directional motion. The conical shape of these tubes can be further exploited for their superior trapping efficiency. Magdanz *et al* used the roll-up tubes to capture sperm and create hybrid micromotors [57]. The trapping efficiency of the tubes might be enhanced by using smaller tubes with conical geometry.

#### 2.2.2. Hybrid tubular motors

**Figure 4.**
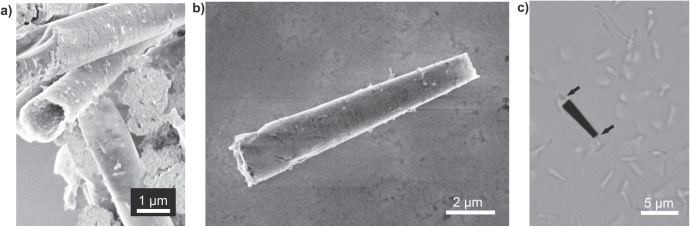
Hybrid microbiorobots. (a) and (b) SEM images of electrochemically fabricated polymer and microtubes, respectively. (c) Bright field image of a biohybrid motor with a single *E*. *coli* bacteria trapped inside a microtube.

In addition to catalytic propulsion, alternative methods for micromotor design should be considered for systems requiring biocompatibility. Biohybrid motors incorporate a living cell into an architecture fabricated from artificial components [58]. Instead of chemically driven micromotor propulsion, which can be toxic to cells, biohybrid motors harvest the mechanical energy of motile cells to drive a motor or perform an assigned task. Biologically driven motors can be externally guided and controlled by biochemical, magnetic, or mechanical stimuli [59]. The electrochemically constructed microtubes of conductive polymers, as seen in figures 4(a) and (b), can be modified with other metals or surface chemistry. The microdimensions of these tubes (5–6 *μ*m long and 1 *μ*m in diameter) offer an ideal frame for smaller cell types, specifically bacteria. Bacteria are capable of multiple types of mobility and are an inexpensive system to convert mechanical motion into controlled propulsion, as they are abundant and simple to culture.

The majority of bacteria-driven motors focus on large numbers of bacteria conjugated to a device to induce motion, but this creates issues with guided cell attachment and controlled directionality. Here, we have addressed this issue by incorporating a nonpathogenic form of *Escherichia coli* (*E. coli*) inside our electrochemically derived polymer tubes (figure 4(c)). These bacteria have multiple flagella for motility, and their small size is advantageous for motors in confined environments. When incubated with a swarm of *E*. *coli*, the polymer microtubes trap single bacteria within the tube for a simple and efficient biohybrid motor. Compared to other cell-tube motor platforms, this biohybrid is more advantageous, as the bacteria occupy the entire tube [57]. If the microtube is much larger in scale than the motile cell, the biohybrid performs as an inefficient motor [57]. As the *E*. *coli* grow to be 5 to 10 *μ*m in length, they are an optimal size for the electrochemically grown tubes. This system has the possibility to be directed with external biochemical cues and can have applications for drug or cargo delivery in biological systems.

#### 2.2.3. Micromotor fabrication using roll-up technology

Tubular architecture helps improve the speed of self-propelled micromotors by bubble production. Microjet-based tubes fabricated using roll-up technology were first reported by Mei *et al* in 2008 [51]. The tubular micromotors are fabricated by the stress-dependent shrinkage and expansion of the nanomembranes [60]. Before growing nanomembranes, lithography patterns are developed on the sacrificial layer on a glass or silicon substrate. Thin films are evaporated on the patterned sacrificial layer, and the evaporations are conducted by electron beam. Shadow angle deposition prevents complete coverage of the sacrificial layer by the evaporated thin film, leaving open windows where the sacrificial layer remains exposed. Nondeposited sacrificial layer windows are important for unidirectional etching of the sacrificial layer. Etching of the sacrificial layer allows nanomembranes to undergo lattice rearrangement to release stress. The first layer, which is typically deposited at the highest deposition rate, starts expanding because of the compressive strain, while the last layer, deposited at a lower rate, starts shrinking to relax the tensile strain. Both effects lead to the nanomembranes rolling up [61]. Rolling-up technology makes it is possible to fabricate micromotors from 1 *μ*m to 30 *μ*m in diameter and 50 *μ*m to 1 mm in length [52].

**Figure 5.**
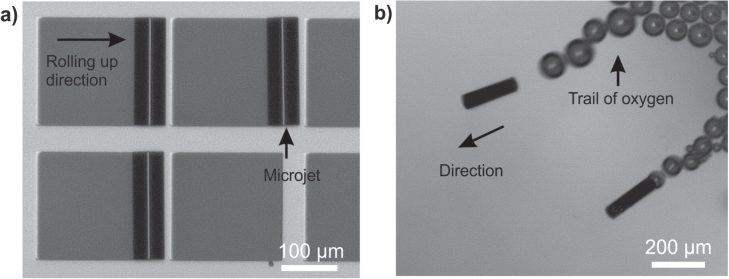
Roll-up fabrication of microjets and self-propulsion. (a) 200 *μ*m-long rolled-up microtube on a patterned glass substrate. (b) Microtubes self-propelling in 10% H_2_O_2_ fuel.

The micromotors have an active catalyst as their top inner layers. The catalyst decomposes the chemical fuels present in the media, and the reaction produces bubbles, which act as propulsion force. Figure 5 shows micromotors fabricated using rolling-up technology and containing Pt as a catalyst, moving in 10% H_2_O_2_ solution. The micromotor has a length of 200 *μ*m and contains three layers of metals deposited at different rates: Fe, 0.8 nm s^−1^; Fe, 0.3 nm s^−1^; and Pt, 0.09 nm sec^−1^. So far, we found roll-up microjets only of 25 *μ*m, 50 *μ*m, and 500 *μ*m in length in previous reports.

### 2.3. Bioinspired motors

Biological systems provide cellular and protein organization to create motors, pumps, and other motilities to remain healthy and survive. By dividing this complex behavior into specific mechanical components, bioinspired motors can be engineered. Techniques such as three-dimensional (3D) printing are implemented to mimic biological systems and provide alternative motors to catalytic particles and tubes.

#### 2.3.1. Sperm-shaped motors

3D printing and stereolithography are easily accessible and inexpensive manufacturing processes that employ polymers to construct layer-by-layer models [62]. Commercially available printers use multiple polymers with different stiffnesses and flexibilities to build complex and organized systems with user-defined pixel sizes. 3D printing can produce objects from macroscale to microscale, with a resolution at ∼16 *μ*m; for this research, we used a compact multimaterial 3D printer (Object260 Connex). This technique allows the fabrication of bioinspired shapes that can be used as motors [63, 64]. These models can help us better understand the mobility and propulsion of microswimmers. An efficient and well-known self-propelled swimmer is the male reproductive cell, the sperm. With its simple structure that includes a single head and tail, it is capable of swimming in different liquid viscosities and against a current. Motion is given by the movement of its tail and directionality is given by its head. The sperm structure can be replicated with 3D printing.

The fabrication process produces a sperm-swimmer made up of two polymers with different stiffnesses, producing a rigid sperm head and a flexible tail. The model that comes out of the printer is covered with a layer of a polymer that is used by the printer as a scaffold structure. This scaffold structure is removed in the last step of the fabrication. The polymer is removed using a 2% NaOH solution. Once the fabrication process is completed, we can give magnetic properties to the sperm-swimmer by depositing a thin layer of iron using an electron-beam deposition process.

**Figure 6.**
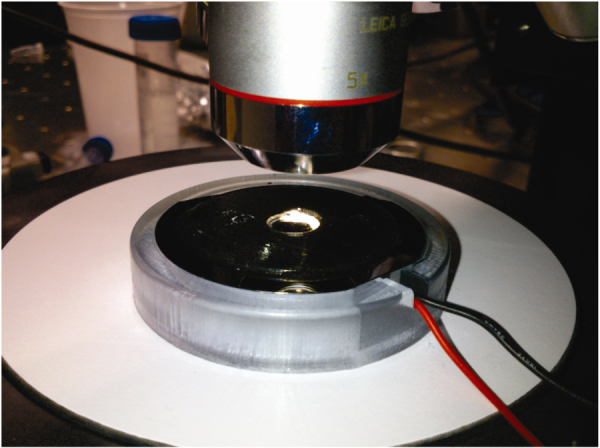
Acoustic set up of a piezo disc with 3.4 MHz resonance frequency, connected to an amplifier and an arbitrary waveform generator.

To generate the motion of the artificial sperm, an external stimulus is provided in the form of acoustic energy. A piezo disc with a resonance frequency of 3.4 MHz is connected to an arbitrary wave generator to introduce a 1 MHz acoustic signal in the system (figure 6). The acoustic stimulation generates nodal fields with high- and low-pressure areas across the system, presenting a unique pattern for each frequency [65, 66].

**Figure 7.**
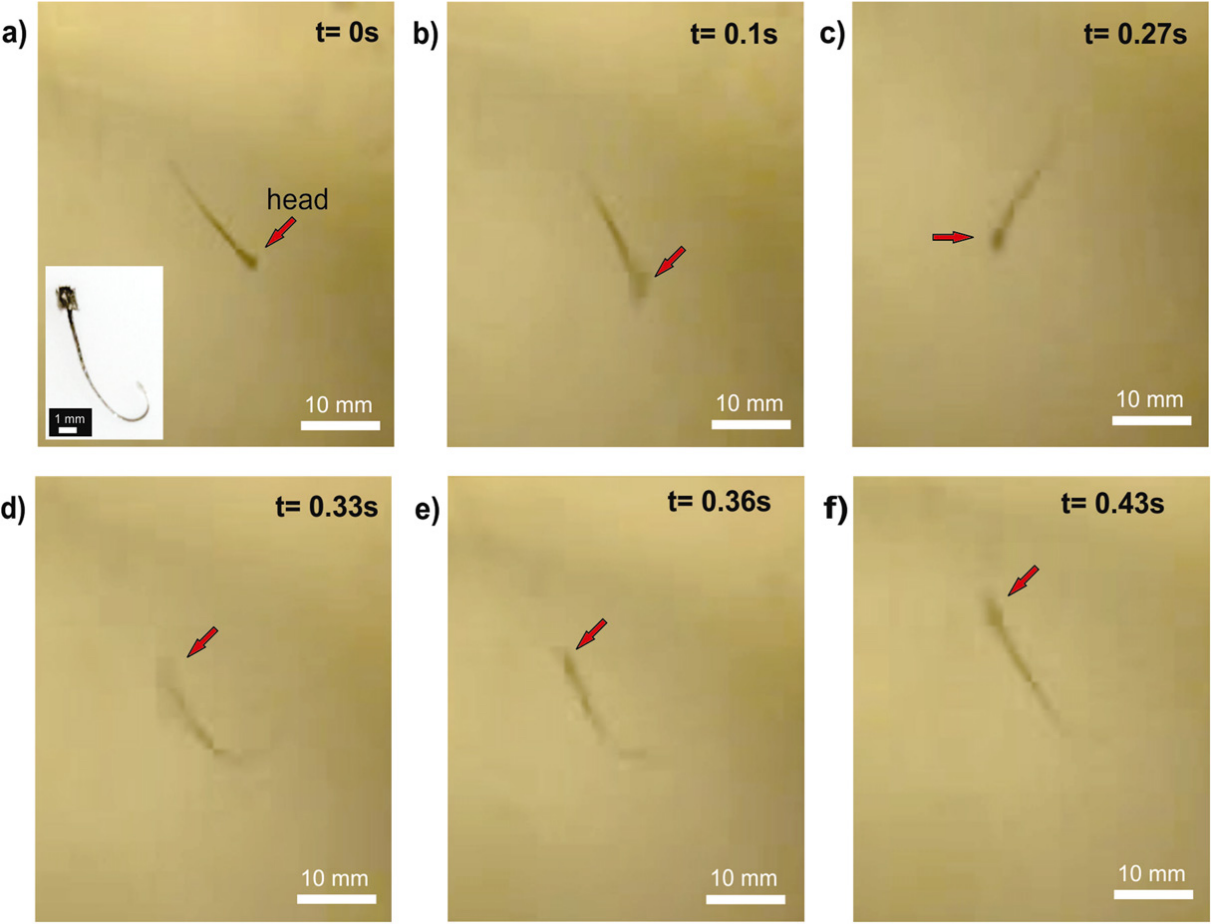
Active artificial sperm. Turn of an acoustically activated sperm-swimmer. (a) No acoustic stimulus. (b) Acoustic energy present in the system felt by the sperm-swimmer. (c)–(e) Sperm-swimmer makes a turn of 180°. (f) Final position.

The nodal fields generated by the acoustic energy activate the sperm and make it move (figure 7). The motion and directionality of the swimmer is dependent on the location of the head [67]. If the head occupies a high-pressure area, the sperm migrates to a low-pressure area. The flexible tail gives an associated drag force that allows the sperm-swimmer to navigate through different high-pressure areas.

As a proof of concept, a sperm-swimmer was activated by an acoustic field of 1 MHz. In figure 7, one can observe 180° rotation of the swimmer in 0.48 s of acoustic stimulus. The future of this research lies in controlling the motion of an artificial sperm-swimmer.

#### 2.3.2. Bioinspired macroscale architecture

As with the previously mentioned microtubes integrated with living cells, macroscale 3D-printed motors can also be fabricated and modified with motile cells. These macroscale systems are often designed and constructed using inspiration from natural biological systems.

**Figure 8.**
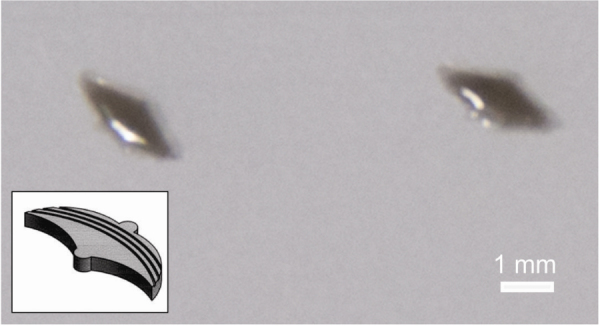
Bat-shaped 3D printed polymer structure for the fabrication of a hybrid motor. Insert shows the schematic drawing of the shape.

The efficient use of energy and motion observed in living species and organisms provides valuable insight into the assembly of artificial motors. Contractile cell types, such as cardiomyocytes or skeletal muscles, offer the mechanical strength needed to power soft robotic systems [68]. These cells’ contractile motion can generate unified propulsion for novel biohybrid motors [64, 69]. The development of 3D printing techniques and materials has generated many opportunities to combine bioinspired architecture with contractile cells. To develop a new macroscale motor, we used a 3D printer to fabricate a winged structure using a flexible, biocompatible polymer. The final printed material is only 2.2 cm in length, and it resembles a bird or bat in its shape, as shown in figure 8. Skeletal muscle cultured on the 3D-printed material can offer a contractile force to propel the material through solution. With an externally applied voltage, the skeletal muscle will contract across the motor surface, pushing the motor forward. In mimicking biological systems, the biohybrid, 3D-printed motors illustrate how a complex behavior can be simplified and used to generate functional motion.

## 3. Outlook

In summary, we have presented an overview of the various fabrication methods to engineer synthetic motors, and we also proposed a new 3D-printing-based fabrication method for the design of bioinspired motors. The concept of guiding Janus motors with walls that are relatively tiny in relation to the size of the Janus motor is reported with preliminary results. We also demonstrated a bioinspired sperm-swimmer’s motion by acoustic energy.

In the future, more research should focus on improving and combining various fabrication technologies to provide synergistic effects and more capabilities to artificial motors. Janus motors will be explored for drug delivery, while microjets may be explored for environmental remediation and other fields where size is not a limitation. More focus will be given to the functionalization of bioinspired shapes, as they hold great promise in the development of biocompatible motors.
